# The effects of COVID-19 on African American communities in Baltimore’s health enterprise zones: a mixed-methods examination

**DOI:** 10.1186/s12889-023-16782-6

**Published:** 2023-09-27

**Authors:** Ludmila F. Wikkeling-Scott, Mohammad Gharipour, Salman Mohagheghi

**Affiliations:** 1 Public Health Department, School of Community Health and Policy, Morgan State University, E. 1700 Cold Spring Lane, Baltimore, MD 21251 USA; 2 Architecture Program, School of Architecture, Planning and Historic Preservation, University of Maryland, 3835 Campus Drive, College Park, MD 20742 USA; 3 Electrical Engineering Department, Colorado School of Mines, 1610 Illinois Street, Golden, CO 80401 USA

**Keywords:** Built environment, Community health, CoVID-19, Health disparities, Health equity, Healthcare access

## Abstract

**Background:**

The CoVID-19 pandemic underscored effects of community resources on the built environment, health and health outcomes. The purpose of this study was to conduct community-engaged research and examine aspects of health, and access to healthcare from the voices of community members, as a foundation for improving health equity through the built environment.

**Methods:**

This study utilized a convergent mixed methods design that included surveys and semi-structured interviews conducted from July 2021 to August 2022 to examine the impact of limited community resources, such as community health clinics on participants during the CoVID-19 pandemic. A convenient sample of 345 male and female African American participants represented five zip codes (21215, 21216, 21217, 21223, and 21229) in with the highest impact from CoVID 19, in Baltimore, Maryland. Quantitative and qualitative data were integrated to describe how the two types supported one another in health, healthcare and healthcare access.

**Results:**

More than half of all participants reported satisfaction with overall health, quality of healthcare provided and access to health care services. However, results indicated extreme differences in factors related to health and wellness after, as comparted to before the onset of the pandemic, Semi-structured interviews, expanded on overall community health, highlighting that overall satisfaction with health does not equal satisfaction with health-related resources and suggested participants felt frustrated and left out of much-needed community health resources to improve health and mental health services for all ages, nutrition services and community activities that make communities thrive. Data integration provided a more realistic view of what participants really experience, due to the expanded analysis of semi-structured interviews, and indicated quantitative and qualitative data did not always support each other.

**Conclusions:**

Future research to improve the built environment, and to address historic health inequities, will require ongoing community engagement to better understand community needs. This study results encourage ongoing research to expand resources for community-engaged research and interventions. Researchers must remain cognoscente of changing needs, and persistent disparities that can only be addressed if policies, supported by these results, are introduced to make equitable investments to forge an environment where healthy communities thrive.

**Supplementary Information:**

The online version contains supplementary material available at 10.1186/s12889-023-16782-6.

## Background

The social and built environments significantly influence public health outcomes, and have great implications for access to care, physical activity and nutrition behavior, [1] housing, [2] and neighborhood safety [3]. These facts became more apparent than ever during the height of the CoVID-19 pandemic in 2020, which disproportionately affected marginalized communities with significant disparities among African Americans, particularly in urban cities [4]. While this is not the first time a pandemic has affected inner cities, little is documented of the non-clinical effects on urban communities prior to the recent CoVID-19 pandemic [5]. In contrast, multiple studies have documented extreme differences in quality of life, morbidity and mortality when comparing persons of different racial and ethnic backgrounds, and of socioeconomic classes [6]. This becomes even more obvious when seen through the lens of their zip codes where health outcomes and wellbeing are directly dependent on locality and affected by health literacy [7]. Policy makers, in an attempt to address such differences, have largely focused on process, administration of finances and delivery of care. The result is that geographical location, environmental conditions, and healthcare access and utilization are a missing link, as is attention to outcomes including life expectancy, infant mortality rates, mental health and substance use statistics, and personal injury [8].

In the United States, racial and ethnic disparities persist, with African American populations experiencing poorer quality of life, and lower access to care [9]. These disparities largely predict health and health-related outcomes, as seen during the CoVID-19 pandemic [10]. In Maryland, the rate of CoVID-19 infections through mid-2022 was higher for African American (49.4%) than for whites (36.9%), Asians (13.7%), and other races [11]. A recent study shows that intermixing morbidity and zip codes has created uneven geographical patterns that particularly impact African Americans. In Baltimore, the thirtieth most populous city in the United States, this is all too visible [10]. Brown (2019) explains that the city’s geographical clustering and structural disadvantages predict health-related outcomes. Policy interventions, like Health Enterprise Zones (HEZ) in Maryland between 2011 and 2014, were implemented to reduce health disparities, improve healthcare access, and health outcomes [12]. More than 80% of HEZ residents self-identify as African American, low socioeconomic status and medically underserved [13]. HEZ interventions have focused primarily on healthcare spending, while ignoring historic segregation that has plagued urban communities and drives poor health outcomes [14]. In the wake of the pandemic, there is consensus that environment and spaces influence disease transmission or facilitation of communicable and non-communicable diseases [15, 16]. This has prompted researchers from various areas of expertise to address the built environment as a critical factor in the provision of care in place, improving health equity and eliminating health disparities [17, 18]. The purpose of this study was to conduct community-engaged research and examine aspects of health, healthcare and healthcare access from the voices of community members, as a foundation for improving health equity through the built environment. For the purpose of this study, healthcare access was limited to a person’s mode of transportation to access health care related services.

## Methods

### Study design

**Fig. 1:**
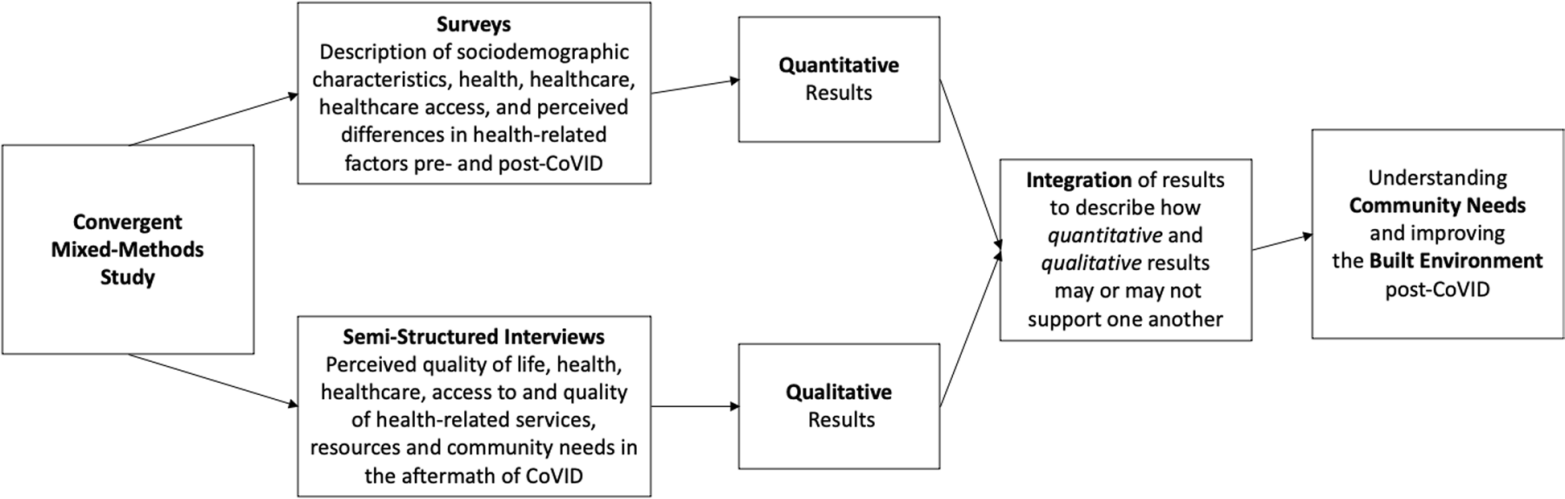
Flow chart of convergent parallel mixed method design to examine effects of CoVID-19 on African American communities in Baltimore City

We used the convergent parallel mixed-method design (Fig. 1) to generate a comprehensive understanding of participant responses, as compared to a single approach [19]. Quantitative and qualitative data were collected and results for each type of data analysis was compared to describe whether the data confirmed each other [20].

Surveys and semi-structured interviews were used to collect data from a convenience sample of residents in the city of Baltimore, Maryland. Five HEZ zip codes—21215, 21216, 21217, 21223, and 21229 were chosen, where CoVID 19 had the greatest impact on residents wo were predominantly African American [21]. To be eligible, respondents had to be 18 years or older and reside in one of these zip codes. Survey and interview questions were designed to determine CoVID-19 related health outcomes including access to care, and community health-related resources. Data for the quantitative strand of this study were collected using community liaisons (CLs), who received a 2-week training on their assigned zip code for survey distribution and completion, to ensure integrity and a positive professional approach towards participants. CLs were provided with a recruitment script for approaching random participants in designated zip codes with Maryland directives for CoVID-19 precautions.

Semi-structured interviews were conducted, for the purpose of this study only, using a participant guide developed by the researchers. Additional research assistants received a 2-day training, to work with community resource centers and recruit participants. Since recreation centers are the main resource for residents in the selected zip codes. Four semi-structured interviews were conducted with community residents recruited from 3 community resource centers, which were the only centers opened after all resource centers were initially closed at the onset of the pandemic. One researcher and two research assistants familiar with the communities and location of the centers, conducted all interviews.

### Procedure

Quantitative surveys, collected over a 1-year period in 2021, consisted of demographic variables, health- and healthcare-related variables, since the onset of the CoVID 19 pandemic. Sociodemographic indicators included age, gender, ethnicity, and education. Additional indicators were height and weight to calculate Body Mass index (BMI) using weight in pounds divided by height in inches squared. Results were categorized by weight status: 0 = underweight < 18.5, 1 = normal weight 185–24.9, 2 = overweight 25–29.9, to 3 = obese 30 or over. Employment, physical activity, whether participants experienced CoVID-19 infection, loss of family members, protective indicators to reduce risk for infection, and aspects of social distancing, were also included. Surveys consisted of 9 Likert-type questions (1 = not at all, 5 = extremely), to measure the association between factors of well-being for pre- and post-CoVID-19 events. Additional Likert scale questions queried factors related to mental health experienced in the two weeks prior to completion of the survey (1 = not at all, 4 = nearly every day).

Qualitative data collection was conducted over a 2-week period in July 2022. Our team conducted semi-structured interviews at recreation centers in collaboration with Baltimore’s Department of Recreation and Parks. Participants were recruited through flyers posted at recreation centers, which assisted in disseminating information via oral and e-mail communication. African American researchers introduced and invited respondents for semi-structured interviews after completing the consent for audiovisual recordings. Each interview was moderated using a semi-structured discussion guide designed by the African American research team. Prompts were added where appropriate, to help elicit feedback on the community’s health and the participants’ experiences accessing community health resources since onset of COVID-19. The guide included prompts to elicit participant responses. Answers were recorded, ranked in order of most important issues, and tallied. All semi-structured interviews were audiotaped and transcribed verbatim. Participants were informed of all study details including their participation in audio recordings, research procedures for confidentiality of data, and use of pseudonyms to maintain anonymity, before completing the consent form, and received $40 gift cards as an incentive for participation in this study. This study was approved by the Institutional Review Board at Morgan State University.

### Data analysis

Quantitative data were analyzed using descriptive statistics, to describe the relationship between demographic variables, health- and healthcare-related variables, since the onset of this pandemic. Researchers tabulated descriptive data to describe socioeconomic characteristics,.i.e.: zip code age, education level, employment status, self-reported height and weight (to calculate body mass index (BMI), and CoVID experience. Likert-type questions were developed to describe participants’ satisfaction with overall health, quality of healthcare and transportation to healthcare services. Participants were further asked to rate differences they experienced in factors related to well-being and functioning pre- and post- CoVID 19 onset, using a Likert-type scale.

Qualitative content analysis, using manually transcribed audio recorded transcripts, was conducted by the researcher and research assistant, trained to interpret qualitative data, to code and identify key themes. Transcripts were analyzed along with field notes. Deductive codes were determined based on the zip code environmental features that promote or discourage equitable health-related resources. To identify emergent themes, inductive coding was performed simultaneously with deductive coding while repeatedly reading the transcripts. The researcher and one research assistant consolidated a list of codes, which were applied to the transcripts. This list was subsequently used to develop themes to describe perspectives among participants. This type of data analysis included identification of words, themes and concepts within the transcripts in the following order: 1) familiarization with the data; 2) generation of codes; 3) search for themes; 4) review themes; 5) defining names and themes. After researchers immersed themselves into the qualitative data, to allow for the emergence of themes, results from both quantitative and qualitative data analysis were compared for similarities and differences. Thus, integrating the data to describe how the two types supported one another in the area of health, healthcare and healthcare access.

This research is based in some of the most marginalized communities in the city of Baltimore, where LSW spent a significant amount of time observing generations of families affected by the instability and lack of community resources. LSW shares time in the area with in-laws who have multigenerational roots for nearly 7 decades in several of the zip codes. Much of the area is governed by policies that have segregated the zip codes from multiple resources that could help communities to thrive, to include community health resources.

## Results

This study was conducted via community-engaged research to address disproportionate access to healthcare and related resources and use of the place-based services as a focal point for improving health outcomes. First, we describe the survey results, followed by a more in-depth look at the community through the lens of residents within the targeted zip codes.

## Quantitative results

### Participant characteristics

Sociodemographic characteristics for survey participants are described in Table 1. Participants were distributed among the following five zip codes: 21215 (71, 20.58%), 21216 (25, 7.25%), 21217 (60, 17.39%), 21223 (67, 19.42%), and 21229 (73, 14.20%).

**Table 1:** Frequency distribution of socio-demographic characteristics

Variable^a^	N	%
Age		
18–25	93	22.57
26–35	97	22.54
36–45	45	10.92
46 or older	62	15.05
Gender		
Male	155	52.73
Female	139	47.28
Ethnicity^b^		
African American	233	67.54
Other	66	19.13
Level of education		
High school level	165	47.83
Undergraduate level	83	24.06
Graduate level	47	13.62
BMI		
Underweight	7	2.03
Normal weight	82	23.77
Overweight	117	33.91
Obese	109	31.59
Employment status		
Employed	220	63.77
Retired	39	11.30
Unemployed	20	5.80
Disabled	1	0.29
Student	31	8.99
Self-employed	2	0.58
You or family ever diagnosed with CoVID		
No	100	28.99
Yes	241	69.86
Ever lost someone due to CoVID		
No	139	40.41
Yes	201	58.43
Primary mode of transportation to health-related services		
Own car	135	39.24
Bus	50	14.35
Walk	30	8.72
Taxi or other	113	32.72
Felt safe when using public transportation		
No	119	34.59
Yes	216	62.79
Zip code you reside in^c^		
21215	71	20.58
21216	25	7.25
21217	60	17.39
21223	67	19.42
21229	73	14.20

*N* = 345

^a^Missing values not included

^b^The target zip codes are predominantly African American

^c^HEZ zip codes 21215, 21216, 21217, 21223 and 21229 (2021)

Participants who reported their age, and were male, included, 57 (36.77%) between the ages of 18–25; 39 (25.16%) between 26–35; 19 (12.26%) between 36–45; and 40 (25.81%) 46 or older, while female participants included 35 (25.55%) between 18–25; 57 (41.61%) between 26–35; 23 (16.79%) between 36–45; and 22 (16.06%) who were 46 or older. In the category of education, less than half of participants had a high school level education, (165, 47.83%); undergraduate education, (83, 24.06%); or graduate level education (47, 13.62%). More than half of participants were employed (220, 63.77%), 39 (11.30%) reported unemployment, and 31 (8.99%) were students, while disabled (1, 0.29%) and self-employed (2, 0.58%) participants were minimally represented. BMI was calculated using self-reported height and weight. Participants were categorized as normal weight, (82, 23.77%); underweight (7, 2.03%); overweight (117, 33.91%); or obese (109, 31.59%).

More than half of the participants reported at least one member in their family, to include themselves, had been diagnosed with (241, 69.86%), and or lost a family member due to CoVID (201, 58.43%). Access to COVID-19 related health services has been reported as important measure of care when symptoms are suspected. Participants’ mode of transportation to CoVID- and other healthcare related services was reported by car (135, 39.24%), by bus (50,14.35%), or taxi (113, 32.72%), while few reported any services in walking distance (30, 8.72%). A number of participants felt safe using public transportation at the time of data collection (216, 62.79%).

### Health, healthcare and healthcare access

The unprecedented events of the recent pandemic highlighted a number of existing disparities in health and healthcare for marginalized communities, and so often the voice of the community is not clearly understood. The researchers chose specific variables from the original survey to describe participant satisfaction with health, healthcare and accessing healthcare. Participants were asked to report on a Likert scale their satisfaction with their overall health (Table 2): very satisfied (102 29.57%), satisfied (184, 53.33%), neither (29, 8.41%), dissatisfied (26, 7.54%) and very dissatisfied (1, 0.29%). Further, participants responded to the quality of healthcare provided in their community: very satisfied (80,23.26%), satisfied (127, 36.92%), neither (86 (25.00%), dissatisfied (33, 9.59%), and very dissatisfied (14, 4.07%).

**Table 2:** Participant satisfaction with factors related to health, healthcare services and healthcare access

Satisfied with:	Very satisfied	Satisfied	Neither	Dissatisfied	Very dissatisfied
Overall health	102 (29.57)	184 (53.33)	29 (8.41)	26 (7.54)	1 (0.29)
Quality of healthcare provided in your community	80 (23.26)	127 (36.92)	86 (25.00)	33 (9.59)	14 (4.07)
Public transportation to access healthcare services	22 (6.40)	75 (21.80)	170 (49.42)	36 (10.47)	31 (2.91)

*N* = 345

For the purpose of this study, health care access refers to a participants’ mode of transportation to accessing healthcare services. Using the same Likert scale, participants responded: very satisfied (22, 6.40%), satisfied (75, 21.80%), neither (170, 49.42%), dissatisfied (36, 10.47%) and very dissatisfied (31, 2.91%).

Using a Likert-type scale, participants were asked to report, notable differences in their physical- and sense of wellbeing in the 4 weeks preceding the survey, as compared to prior to the inception of the COVID-19 pandemic. More than half of all participants reported extreme differences in each of the following categories: ability to concentrate (190, 61.49%), being productive (163, 52.92%), sleep in (183, 60.60%), feeling lonely (183, 60.00%), being depressed (184, 60.13%), being angry (202.66.23%), and experiencing grief (215, 70.26%). Participants who experienced extreme differences in being bored (145, 47.39%) and being frustrated (148, 48.37%) represented almost half in these categories (Table 3).

**Table 3:** Perceived differences experienced in factors of wellbeing and functioning pre and post -CoVID-19^a^

	Extremely	A lot	Moderately	A little bit	Not at all
Experienced differences:					
Concentrating	190 (61.49)	68 (22.01)	26 (8.41)	18 (5.83)	0 (0.00)
Being productive	163 (52.92)	78 (25.32)	45 (11.36)	23 (7.47)	9 (2.92)
Being Bored	145 (47.39)	69 (22.55)	42 (13.73)	26 (8.50)	24 (7.84)
Sleeping in	183 (60.60)	71 (23.51)	17 (5.63)	24 (7.95)	7 (2.32)
Being lonely	183 (60.00)	65 (21.31)	21 (6.89)	25 (8.20)	11 (3.61)
Being depressed	184 (60.13)	60 (19.61)	25 (8.17)	23 (7.52)	14 (4.58)
Being frustrated	148 (48.37)	52 (16.99)	32 (10.46)	44 (14.38)	30 (9.80)
Being angry	202 (66.23)	50 (16.39)	22 (7.21)	23 (7.54)	8 (2.62)
Experiencing grief	215 (70.26)	45 (14.71)	15 (4.90)	22 (7.19)	9 (2.94)

*N* = 345

^a^Pre-CoVID is considered before the pandemic ensued, while post-CoVID is considered the 4 weeks after the pandemic

While quantitative results provided some information about the participants’ health and well-being and CoVID-19 related experience, the semi-structured interviews revealed more in-depth self-analysis. During these interviews, participants described the community’s access to healthcare and related services, and the healthcare infrastructure as it currently exists. Upon analysis of qualitative data, the authors intend to describe how results compare to qualitative results around health, health care and health care access.

## Qualitative results

### A closer look at community health and healthcare services

A total of 25 African American participants met the criteria for semi-structured interviews related to community health and healthcare services, and included both males (8, 32%) and females (17, 68%) Among participants who reported their age, 4 (16%) were between 18–25, 2 (8%) between 26–35, 7 (28%) between 36–45, 12 (48%) 46 or older. When asked about their current access to health care benefits, 4 (16%) indicated they had employer-provided benefits, 1 (4%) was uninsured, 12 (48%) received Medicaid, 8 (32%) received Medicare, ad 2 (8%) received Veterans health care benefits.

Qualitative questions were designed to gain a better understanding of perceived quality of life, community health, present healthcare resources, and participants’ expectations for future community resources used to improve the built environment. Analysis of the participants’ transcripts revealed five main themes: a sense of community, lack of community resources, food desert/lack of healthy food, lack of quality healthcare, mental health needs, and youth at risk (Table 4).

**Table 4:** Themes and direct quotes from participants across five zip codes in the city of Baltimore

Theme	Participant responses	Description
Sense of Pride	Just to say that my kids are still here, and they are here, live in the city of Baltimore I’m proud of, of course, I would like to see some change in Baltimore, we will all have to come like together as a whole ~ Female Participant (Bentalou Recreation Center) I was born and raised just a little bit I was born and raised in the time of Jim Crow. I’ve seen a lot of history and my family was involved in a lot of history like several magazines … a lot, a lot of good people who we look up to like Thurgood Marshall, I mean, it’s a lot of people know just about you know, being on an avenue and things, their lifestyle has taught me, that things they said we couldn’t do, we couldn’t be, we could be ~ Male Participant (Crispus Attuck Recreation Center) I was born and raised here, and I'm proud of my city because they have like some great school for the kids and also as the part of our Baltimore recreation and parks we got a lot of programs to do for the kids so they don’t have to get involved in a lot of hustle activities ~ Male Participant (Crispus Attuck Recreation Center)	A sense of pride A sense of accomplishment and historic value A sense of belonging
Lack of community centers/ resources	I want to see some health resources, where there was no charge. Get the folks who have no transportation, take them to these different locations to participate in these different things ~ Female Participant (Bentalou Recreation Center) Because some places they got extra trains to get to it traveling, everybody don’t have transportation, everybody don’t have $5 and 50 cent or whatever it costs to get on the bus ~ Female Participant (Bentalou Recreation Center) In my community, I wish there were like stores or things closer to me that I can walk to you because I really like walking and exercise ~ Male Participant (Bentalou Recreation Center) I went to school around here, when I was 17 I was in marching bands, they had rec centers, they had pools, play grounds, but they took all the main buildings away, like they took one of our community centers and turned it into a shelter, not saying that’s a bad thing, but nobody in the neighborhood had nowhere to go ~ Male Participant (Bentalou Recreation Center) It is really nothing in the community to raise the kids. And see I grew up doing outreach, there is nothing to do like outreach. There is no stuff for young people ~ Male Participant (Crispus Attuck Recreation Center) You don’t put a community health center in a broke community and then try to charge them. At least have some free services that people can use. Everybody ain’t got insurance, everybody can’t afford a copay ~ Male Participant (Crispus Attuck Recreation Center)	A sense of being overlooked A sense of a lack of government consideration A sense that the leadership does not care enough to invest in their communities
Lack of quality care and mental health services	Man they gave me some medicine that made me chipper, as fast as I passed it out, as fast as I threw it away ~ Male Participant (Crispus Attuck Recreation Center) I got a whole lot of prescriptions at home stacked up in the drawer, and I know I can take that on the corner, and in a month or so after somebody take it … you know what I mean … no I ain’t gonna do that. I am just saying, there will be many more killings out there than there is now ~ Male Participant (Crispus Attuck Recreation Center) That’s bad, the mental health … that’s bad … there’s people out here that you don’t know– the very person you walk by could have issues, be mentally unstable, you don’t know and a lot of things that’s going on a lot of things people are doing, mentally they’re not there. What are they going to do and what services are really offered for those people? The mental health situation in this city is bad ~ Female Participant (Crispus Attuck Recreation Center) From my doctor, the internet, 21,217 really don’t have no services no more. Everything they had turns into something else. They have a bunch of empty lots, junkies ~ Female Participant (Bentalou Recreation Center) I mean, having health insurance is a concern; I think violence being addressed, drugs and violence is a concern; I think … mental health needs need to be addressed because there is a lot going on and a lot of people are undiagnosed unless the drugs come in with the self-medication and all of that ~ Female Participant (Bentalou Recreation Center)	Concern for excessive prescription drug use Concern for rising mental health issues without access to mental health services
Food desert/ Lack of healthy food	In my community there used to be a market. I used to like it, I stayed in that market and would go shopping in a minute. When COVID came they shut it down ~ Male Participant (Crispus Attuck Recreation Center) Places should have a food pantry, for people who can’t afford to get it for their kids and stuff … I feel like if they can make a big community center of everything that somebody needs in the community and have a good hand of people that works there ~ Female Participant (Bentalou Recreation Center) So, for me is eating healthier. Because these kids do not understand what good food is. The food that we get here now is this unhealthy food ~ Female Participant (Bentalou Recreation Center) The poor food habits, that would be something where they just don’t tell us but they actually show us ~ Female Participant (Bentalou Recreation Center)	A sense of being excluded from healthier food access Lack of quality food throughout the community Concern for health consequences as a result of the lack of quality foods No nutrition programs to create awareness about healthier lifestyles
Youth at risk	You see, you see a basketball court right here, but a lot of them have been torn down. So it's like it's not like kids can go … is there’s nowhere for them to go but outside. The swimming pool? No, they just saw open up some of the pools, you just got to follow the rules ~ Male Participant (Bentalou Recreation Center) When I drop of my kids and I gotta go in there and remove a needle from the playground … and they’re getting them from hospitals, soon as they get the opportunity they go to the hospitals and get these syringes, going to sell them, going to use them ~ Female Participant (Bentalou Recreation Center) There is a whole bunch of stuff out here, this is stuff you get from a psychiatrist and most of them people) out here getting high, and most of the young kids that have died in the last couple of years, most of them if they didn’t get shot it was from overdoses from pills. Not from crack cocaine, dope, no it was mainly from pills. So they do a lot of pill usage, there are still people who are getting high but it is mostly the pills. I think they sell more pills than they sell anything ~ Female Participant (Bentalou Recreation Center) I don't feel I don't feel too safe, but I just be aware of my surroundings. Because a lot of what's going on is beyond our control. What can you do? ~ Male Participant (Crispus Attuck Recreation Center)	The environment for youth is impacting the community, due to the lack of youth resources and programs Lack of community resources increased the lack of youth programs and subsequent negative behaviors Health care providers’ carelessness in prescribing drugs leads to too many prescribed pharmaceuticals becoming readily available on the streets

### Sense of community

Throughout the participant interviews, we noted a sense of community, and a strong sense of pride that was based on historical accomplishments and notoriety established in the city of Baltimore. Throughout the interviews, participants shared a sense of satisfaction with their current place of residence because of their pride in the city, while expressing concerns for community needs. It is important to note that participants did not suggest that they wanted to leave their community, they just wanted to improve their community, with help from outside. Issues of safety, while affecting their satisfaction, did not change their sense of pride in being a resident of the city. Their place in the community was important and helping each other, knowing they have each other gave them a sense of self-empowerment.

### Community centers and resources

Concern for lack of community centers and resources arose from combining similar categories, including: a lack of family programs; lack of fitness programs; lack of elderly care programs; lack of healthcare specialists; lack of mental health services (especially after CoVID-19. Residents felt they needed more mental health services in the community, so that persons would not have to travel and pay for transportation to meet their needs. Other categories include lack of: preventive care; adult programs; youth programs; food pantries; feeling forgotten; community programs; quality care; and adequate waste management. Participants agreed that there is a lack of investment in their community and an unwillingness to pay attention to dire needs. Participants noted government programs overlooked and underestimated their needs, as if “they don’t care”. Expanding on this theme of “they don’t care” were expressions that suggested the government could come into communities to shut or tear down resources, stores, and children’s playgrounds to benefit government officials and developers, but not necessarily the community.

### Access to nutrition and the lack of healthy food

The theme, lack of healthy food options, provided important information about participant’s feelings that they were in the “wrong zip code” and excluded from nutrition services that would provide them with healthier food options. This represents deep-rooted racially influenced concerns about negative perceptions for certain zip codes and lack of investment. Participants compared their zip codes to South Baltimore zip codes, where community centers provide daily meals, assistance with utilities, and transportation so that members of the community can survive and thrive. Yet, in their own area, they described grocery stores closing, with no alternatives for accessing healthy food options, leaving them helpless regarding nutrition habits. The quotes presented demonstrate lack of investment in sustainable resources that improve health outcomes, and a lack of investment towards a healthier community.

### Quality care and mental health services

The overarching theme, lack of quality healthcare, was consistent throughout all participant responses. During the height of CoVID-19 lockdown, participants experienced more obvious mental health problems due to loss of employment, lack of employment opportunities, and a feeling that healthcare providers were out of touch with community needs. Participants agreed that healthcare providers were eager to write prescriptions for mental health conditions leading to prescribed medications falling into the hands of youth for whom they were not intended. Some participants shared how they found out their healthcare providers no longer accepted their insurance after CoVID-19, with no alternatives for follow up coverage, affecting adherence to care, and increased the potential for self-harm and suicidal behavior. One participant shared that he attempted suicide because he could not figure out how to care for his family. Some male participants described a strong urge to protect their community, since no one seemed to care that these issues were negatively affecting their daily lives, sense of safety, and sanity. Participants suggested that patterns of community health were not due to their lack of awareness about obesity, sedentary lifestyle, and poor nutrition, but rather a lack of resources, opportunity for physical exercise, a sedentary lifestyle due to unemployment, and the feeling that health care providers were just pushing prescriptions without empathy for the patient.

### Youth services and at-risk youth

For the purpose of this study, at-risk youth refers to youth engaged in substance use and violence due to a lack of neighborhood resources to positively engage their age cohort. In one zip codes, once home to Thurgood Marshall, Billie Holiday and Cab Calloway, rates of unemployment, crime and poverty are well above the city’s average, and participants support previous reports of a community in despair [22]. Previous studies suggest that food insecurity, poor nutrition and reduced physical activity can impair cognitive development, and increase mood and behavior changes [23]. Participants expressed deep concern for the future of their youth, perceiving a limited potential for accomplishments due to a lack of youth programs, the recent closing of recreation centers, and the increasing ease of access to prescription drugs. Neighborhood investments influence youth health outcomes, as one participant suggested. There was consensus that “we must pay attention to our youth, or they will become a lost population: youth will not be able to thrive if programs are not put in place.”

## Discussion

The results from this mixed-method study reflect the HEZ communities in Baltimore most affected by the CoVID-19 pandemic. There is an urgent need for improvements to make community centers, parks, markets with healthy food, and healthcare facilities accessible to address community health and wellbeing on multiple levels. Participant responses suggest a socially vulnerable and marginalized community. A general observation was a low level of health literacy based on participant input during semi-structured interviews, and the lack of understanding of basic terminology related to health and wellbeing. The dimensions of health, healthcare infrastructure, health care access and the need for addressing health literacy, will be the focus of this discussion.

### Health and healthcare infrastructure

Overall, participants felt a sense of pride in their community. Survey results showed most participants are satisfied with their overall health but desire a vested interest in the improvement of health-related resources to improve health outcomes. While quantitative results suggest an overall satisfaction with healthcare services, a more in-depth look at the healthcare infrastructure revealed a strong need for more. Participants discussed their dissatisfaction with the current available healthcare resources and gave authors a more extensive interpretation of their satisfaction or lack thereof with current community health resources. Participants shared examples to drive the point that their communities were marked by lack of investment in resources, diminishing services for youth and adults, and reduced community programs to support a healthy and positive community environment.

While quantitative results were limited to a few indicators of participant satisfaction, the semi-structured interviews allowed the authors to recognize some differences between the two types of results. Participants may feel an overall satisfaction with what they currently receive, but this should not be interpreted as an overall satisfaction with health. This is important for future interventions that may possibly limit their scope based on merely quantitative responses that do not paint an accurate picture of the reality on the ground. This was further shown by the differing results between quantitative data on participant satisfaction and expanded discussions which painted a clearer picture. Participants felt that the lack of health-related infrastructure added to adverse health conditions, and a sense of powerlessness.

### Healthcare access

While survey participants expressed a more neutral satisfaction with transportation to access healthcare services, semi-structured interviews revealed a different view. Participants expressed that most healthcare services were outside of their zip codes, and that CoVID-19 caused a decrease in existing services, further diminishing their access to healthcare. While it may be neither satisfactory or dissatisfactory to use public transportation and access healthcare, there is a bigger issue: health care services that are located mainly outside of the community create additional barriers to overall health as services also become more limiting. This was expressed when participants discussed the need for mental health services in the community. Mental health concerns, as expressed during semi-structured interviews, were exacerbated by lack of quality services, and transportation. Factors such as education, community programs for all ages, and recreational programs with positive activities and outreach for youth, also needed safe and reliable transportation which is currently not accessible or affordable by all. These experiences an observations are in line with multiple studies that have documented social disparities and inequities affecting African American communities nationwide [24].

### Communication about health and healthcare

Survey participants expressed notable differences in factors related to well-being pre- and post-CoVID 19 onset, which was further explained and supported by semi-structured interview results. Participants expressed their increased frustration with health-related services and infrastructures that are currently not conducive to address their health and mental health needs. Participants discussed the obvious increase in health- and mental-health symptoms in need of ongoing services. Quantitative and qualitative results complement one another to better understand the needs of these HEZ communities. In the presence of the increasing changes, particularly in mental health-related factors, participants related that there were challenges in communication and understanding between patient and physician, indicative of a lack of health literacy.

Although health literacy was not included in this study, it cannot be ignored as researchers attempt to describe the responses from community-engaged activities such as the surveys and semi-structured interviews. Probing questions posed by researchers sometimes resulted in confusing responses that were indicative of the varied levels of understanding about terms such as “quality of life,” “community health,” “healthcare access,” “community health services,” “healthcare provider,” and “primary health care.” Additional probing terms were sometimes used to clarify for participants what the researchers were asking. Throughout each semi-structured interview, participants remained eager to share their stories and describe the impact of CoVID-19 on their community.

Quantitative and qualitative results did not fully support one another, yet findings from the qualitative results are supported by previous research [25] on how HEZ communities are negatively affected by a lack of government investment in the community’s overall wellbeing [26]. Persistent structural racism is a major factor in this negative affect and investment response [27]. There is much room to expand this research on the impact of COVID-19 on marginalized communities that stretches beyond their access to care to include place-based services and geographical impact on health and wellness.

By partnering with communities to understand historic health disparities, further highlighted in the era of CoVID 19, this study identified patterns of health inequities that negatively affect health outcomes and provide fuel for ongoing disparities. The results of this study revealed that the barriers to community health infrastructures to address health and health-related needs are the result of age-old urban policies, and an urgent need for change and immediate action. An environment that lacks a healthy infrastructure cannot result in healthy outcomes.

### Strengths

First, the strength of this study was the community-engaged approach to understanding health, healthcare and health care access needs from the eyes of the target population, thus lending a voice to the often voiceless. Second, the data was collected by persons familiar with the community, experienced the community and its struggles and was representative of the racial and ethnic makeup of the target community to address cultural sensitivity. Third, the interpersonal communication between participants during the semi-structured interviews helped to clarify differences in expressed opinions and values.

### Limitations

This study has many strengths, but the analysis was based on data from a limited number of participants. First, the authors used convenience sampling, which may be highly efficient but can lead to an underrepresentation of some groups within the study sample. Therefore, the convenience sample may not be fully representative of the population of interest and influence the results. Second, participant experiences may have depended on where participants received care and may have differed according to education and employment status. Third, in the absence of a piloted survey, validation of survey tools, and lack of familiarity with some terminology used during semi-structured interviews, this may have influenced participant responses and analysis of results.

## Conclusions

Community engagement is critical for improving the built environment in researchers’ efforts to correct health inequities. Traditional quantitative surveys do not necessarily provide a true explanation of health conditions, perceptions, barriers, and outcomes, as seen in this study. Some results are supported, while others are not, when additional input is provided through semi-structured interviews. Prior to the CoVID 19 pandemic, studies warned about health inequities across a wide range of social and health dimensions, creating trends that disproportionately affected communities with lack of resources and investments, and where African Americans were concentrated in inner cities [28]. Disparities that arose during the CoVID 19 pandemic further highlighted these inequities. Often communities like those identified for this study, find themselves so far behind in public and private investments to infrastructure and pertinent resources, that small actions on the part of a few do very little to correct age-old policy-driven inequities negatively affecting health and health outcomes. Therefore, the results of this study serve to encourage ongoing research and to expand resources for community-engaged research and interventions. Researchers must remain cognoscente of changing needs, and persistent disparities that can only be addressed if policies, supported by these results, are introduced to make equitable investments to forge an environment where healthy communities thrive. This should not be the desire of a few, but rather of a collective community where everyone’s health and health outcome are seen as a benefit to all, rather than the privilege of a few.

### Supplementary Information

**Additional file 1.** Semi-structured interview – questionnaire.

## Data Availability

The datasets generated and analyzed during the current study are not publicly available but are available from the corresponding author on reasonable request.

## Abbreviations

BMI: Body Mass Index
CoVID-19: Coronavirus disease 2019
CLs: Community Liaisons
HEZ: Health Enterprise Zone
U.S.: United States
