# Supplementary material for: The effects of COVID-19 on African American communities in Baltimore’s health enterprise zones: a mixed-methods examination
Source: BMC Public Health. 2023 Sep 27;23:1873. doi: 10.1186/s12889-023-16782-6 (PMC10536730; doi:10.1186/s12889-023-16782-6)
Supplement: Supplementary file 1 — Additional file 1. Semi-structured interview – questionnaire. [file 12889_2023_16782_MOESM1_ESM.pdf]

SEMI-STRUCTURED INTERVIEW - QUESTIONNAIRE

The Effects of COVID-19 on African American Communities in Baltimore's Health Enterprise Zones: A Mixed-Methods Examination

---

Protocol

Thank you for completing the consent form and for filling out the questionnaire to give us some idea of your background and health experience. Now we are moving on to the semi-structured interview section as discussed in our introduction.
For the purposes of this discussion, "community" is defined as the place where you live, come to after work, and your children play.

Opening Question (5 minutes)

1. To begin, why don't we go around the table and introduce ourselves. Please tell us your name (or how you would like us to call you) and then share something that makes you most proud of the Baltimore community you live in.

I want to begin our discussion today with a few questions about health and quality of life in your community. What I mean by that, how satisfied are you with your family life, your job, your social life, spiritual life, your overall health and safety?

2. What does quality of life mean to you?

3. Now that we have discussed a little bit about quality of life, let's go a step further. In your mind, what makes your community healthy?

4. Can you tell us a little about who the healthy people are in your community?
    - 4.1. What makes them healthy?
    - 4.2. Why are these people healthier than those who have (or experience) poor health, and what makes them healthy?

5. Let's take a look at your community. What do you believe are the 2-3 most important issues that must be addressed to improve health and quality of life in your community? [Prompt]
    - 5.1 What are the biggest health problems/conditions in your community? Family Questions (20 minutes) Now we are going to transition a bit and focus a bit more on your family and experiences.

Family Questions (20 minutes)

Let's talk a little bit about your family experiences.

6. What types of services or support do you (your family, your children) use to maintain your health?
    - 6.1. Why do you use these particular services or supports?
    - 6.2. How satisfied are you with the quality of the health services offered in your community?

6.3. What about mental health services, are there any offered in your community?
6.4. How satisfied are you with the quality of the mental health services offered in your community?

7. Where do you get the information you need related to your (your family's, your children's) health?
7.1. What other health resources do you rely on in your community?
7.2. If a new community clinic were set up in your neighborhood, what would you like to see offered as services to the community?

8. What keeps you (your family, your children) from going to the doctor or from caring for your health? [Prompts]

8.1. Are there any cost issues that keep you from caring for your health? (Such as co-pays or high-deductible insurance plans)
8.2. If you are uninsured, do you experience any barriers to becoming insured?

Improvement Questions (20 minutes)
Next, I'd like to ask a few questions about ways to improve community health.

9. Do you have some ideas for a community health center to help your community get or stay healthy?

10. What else do you (your family, your children) like to see in a community clinic, to maintain or improve your health? [Prompts] What about…
10.1. Information to manage a chronic condition or information to change your health behaviors such as poor food choices, smoking, eating habits, physical activity, or substance use?
10.2. Services to prevent illness and poor health outcomes, access to immunizations?

11. What resources does your community have at the moment, that can be used to improve community health? Ending Question (5 minutes)

12. Is there anything else related to the topics we discussed today that you think I should know that I didn't ask or that you have not yet shared?

Facilitator Summary & Closing Comments (5-10 minutes)
Let's take a few minutes to reflect on responses you provided today. We will review the notes we took and the themes we observed. This is your opportunity to clarify your thoughts or to provide alternative responses.

[Co-facilitator provides a brief summary of responses for each of the questions or asks clarifying questions if she thinks she may have missed something.]

Thank you for your participation in this semi-structured interview. You have all raised several great issues for us to consider. We will look at what you have told us and use this information to make recommendations to create healthier communities.

SEMI-STRUCTURED INTERVIEW - QUESTIONNAIRE

The Effects of COVID-19 on African American Communities in Baltimore's Health Enterprise Zones: A Mixed-Methods Examination

---

Participants are asked to fill out this demographic questionnaire to describe some factors that may affect the qualitative results:

QUESTIONS	ANSWERS	
Please provide your age range	☐ 20-29<br>☐ 30-39<br>☐ 40-49	☐ 50-59<br>☐ 60 or older
Please identify your race and ethnicity	☐ American Indian/Alaska Native<br>☐ Asian Pacific Islander	☐ Black/African American<br>☐ Hispanic/Latino<br>☐ White
Gender	☐ Male<br>☐ Female	Transgender<br>Identified as LGBTQ
What is your marital status?	☐ Married<br>☐ Widowed, Separated, or Divorced	Never Married<br>Living with Partner
How many persons live in your household?		☐ 1 ☐ 3-4<br>☐ 2-3 ☐ 4 or more
Do you know the average size of households on the street where you live?		☐ 1 ☐ 3-4<br>☐ 2-3 ☐ 4 or more
Are you a parent of a child under 18	☐ No	Yes
What is your education level?	☐ No educational degree<br>☐ High School degree<br>☐ Some College<br>☐ Completed College	☐ Some Graduate college<br>☐ Master level graduate<br>☐ Doctoral level graduate<br>☐
Do you have health insurance	☐ Employer - provided<br>☐ Directly purchased<br>☐ Uninsured	☐ Medicaid<br>☐ Medicare<br>☐
Have you ever experienced any of the following? (Check all that apply)	☐ Diabetes<br>☐ Obesity<br>☐ Heart Disease<br>☐ Cancer	☐ Surgery<br>☐ Hypertension<br>☐ Dental Disease

QUESTIONS	ANSWERS	
Has anyone in your family other than yourself, ever experienced any of the following (check all that apply)	☐ Diabetes<br>☐ Obesity<br>☐ Heart Disease<br>☐ Cancer	☐ Surgery<br>☐ Hypertension<br>☐ Dental Disease<br>☐
Do you use public transportation to visit health care services, such as a doctor's office, clinic, or hospital?	☐ Yes, always<br>☐ Yes, sometimes<br>☐ No, never	
How safe do you feel when using public transportation?	☐ Very safe<br>☐ Safe<br>☐ Neutral<br>☐ Unsafe<br>☐ Very unsafe<br>☐ I do not use public transportation	
